# Comparison of self versus expert-assisted feedback for cricothyroidotomy training: a randomized trial

**DOI:** 10.1186/s12909-022-03519-z

**Published:** 2022-06-14

**Authors:** Hasan Aldinc, Cem Gun, Serpil Yaylaci, Cigdem Ozkaya Senuren, Feray Guven, Melike Sahiner, Kamil Kayayurt, Suha Turkmen

**Affiliations:** 1 Department of Emergency Medicine, School of Medicine, Acibadem Mehmet Ali Aydinlar University, Istanbul, Turkey; 2 Department of First and Emergency Aid, Vocational School of Health Services, Acibadem Mehmet Ali Aydinlar University, Istanbul, Turkey; 3 Center of Advanced Simulation and Education (CASE), Acibadem Mehmet Ali Aydinlar University, Istanbul, Turkey; 4 Department of Medical Education, School of Medicine, Acibadem Mehmet Ali Aydinlar University, Istanbul, Turkey; 5 Emergency Department, Hamad Medical Corporation, Doha, Qatar

**Keywords:** Medical education research, Simulation, Feedback

## Abstract

**Background:**

The self-video feedback method may have the potential to provide a low-cost alternative to physician-driven simulation-based training. This study aimed to assess the utility of two video feedback methods by comparing the improvement in performing cricothyroidotomy procedure following self video feedback (trainees review their performance by themselves) and expert-assisted video feedback (trainees review their performance while an emergency physician provides additional feedback).

**Methods:**

This study was pretest-posttest and two-group designed research performed at a university simulation center with 89 final-year medical students and used a cricothyroidotomy simulation model. After seeing an educational presentation and a best practice video, trainees were randomized into two groups; self video feedback group (SVFG) and expert-assisted video feedback group (EVFG). They performed the cricothyroidotomy before and after the feedback. The procedures were also recorded and scored by two emergency physicians.

**Results:**

There was a statistically significant improvement between pre-feedback and post-feedback assessments in terms of scores received and time needed for the procedures in both SVFG and EVFG groups (*p* < 0.05). Additionally, the post-feedback assessment scores were higher and time needed for the procedure was lower in the EVFG when compared with SVFG (*p* < 0.05 for both).

**Conclusions:**

Results demonstrated significant improvement in cricothyroidotomy performance with both types of video feedback method. Even though the improvement was better in the EVFG compared to the SVFG, the self video feedback may have value especially in situations where expert-assisted feedback is not possible.

**Supplementary Information:**

The online version contains supplementary material available at 10.1186/s12909-022-03519-z.

## Introduction

In medical education, simulation-based training is a rapidly expanding field due to advances in technology [1]. Simulation-based medical training aims to create real life-like situations using specifically-designed, structured systems. In this simulated environment, students can improve their skills, knowledge, and capabilities [2].

Feedback in simulation-based training is defined as “specific information that compares a trainee’s performance to the preset standard, aiming to improve the trainee’s performance” [3]. It is an important process that stimulates the trainees to reflect on their performance [3]. Despite the established importance of feedback in academic literature, enabling effective feedback remains difficult to achieve in the area of clinical training [4]. Failure at providing effective feedback to medical students has been widely discussed in recent years [5]. Feedback provided being overly complex, delayed, or overly brief were some of the common complaints coming from the students [5, 6].

As an alternative way to evaluate performance, video feedback (viewing the video recording of one’s own performance) may improve quality of the debrief and enhance learning [7]. Additionally, self-assessment has been long known as an important method in the development of learning skills [8]. Different names such as ‘self-directed model’ or ‘learner-centered education’ have been used for self-assessment or self-education-based methods. It has been stated that the lack of ability of learners to assess themselves is one of the main issues related to this method, and it strongly depends on various factors and circumstances. Directed self-guidance is a somewhat-modified version of these methods, in which there is additional educational content to aid the self-learners [9]. Also, it was observed that students demonstrated better psychomotor skills via self-feedback [10]. However, it has been stated that self-evaluation methods may be limited by the the trainees’ inadequate knowledge and experience [7].

A randomized study revealed that expert-assisted feedback provided no additional advantage over self video feedback [10]. In contrast, Vnuk et al. reported that students viewing the video recording of their performance did not improve the agreement of trainees’ self-assessment with the objective assessment by the expert [11]. In light of these findings, the self video feedback method can be expected to reduce the need for the feedback from an expert during simulation-based training, and may have the potential to provide a lower-cost alternative to expert-assisted version.

Emergency cricothyroidotomy is a potentially life-saving procedure performed to provide oxygenation if neither intubation nor a mask or mouth-to-mouth ventilation is possible [12]. However, cricothyroidotomy may not always be performed successfully [13]. Its life-saving potential necessitates training and experience for clinicians [14]. The training of students on this procedure is mostly simulation-based [15].

This study aimed to assess the utility of two video feedback methods by comparing the improvement in performing cricothyroidotomy procedure following self video feedback (trainees review their performance by themselves) and expert-assissted video feedback (trainees review their performance while an emergency physician provides additional feedback). We used “Emergency Cricothyroidotomy Simulation” training on a realistic mannequin model.

## Material and method

### Study design

This randomized educational intervention study was performed between September 2019 – November 2019 at Acibadem Mehmet Ali Aydınlar University Simulation Center, Istanbul, Turkey.

### Sample and sampling method

All final-year medical students at the Acıbadem Mehmet Ali Aydınlar University were invited to participate in the study as trainees. The trainees had no previous training on performing cricothyroidotomy. The study protocol was approved by the Acıbadem Mehmet Ali Aydinlar University Medical Research Ethical Committee (ATADEK-2019/14).

The trial was registered to ISRCTN Registry Service on 16/05/2022. Registration mumber: ISRCTN35998024.

**Fig. 1:**
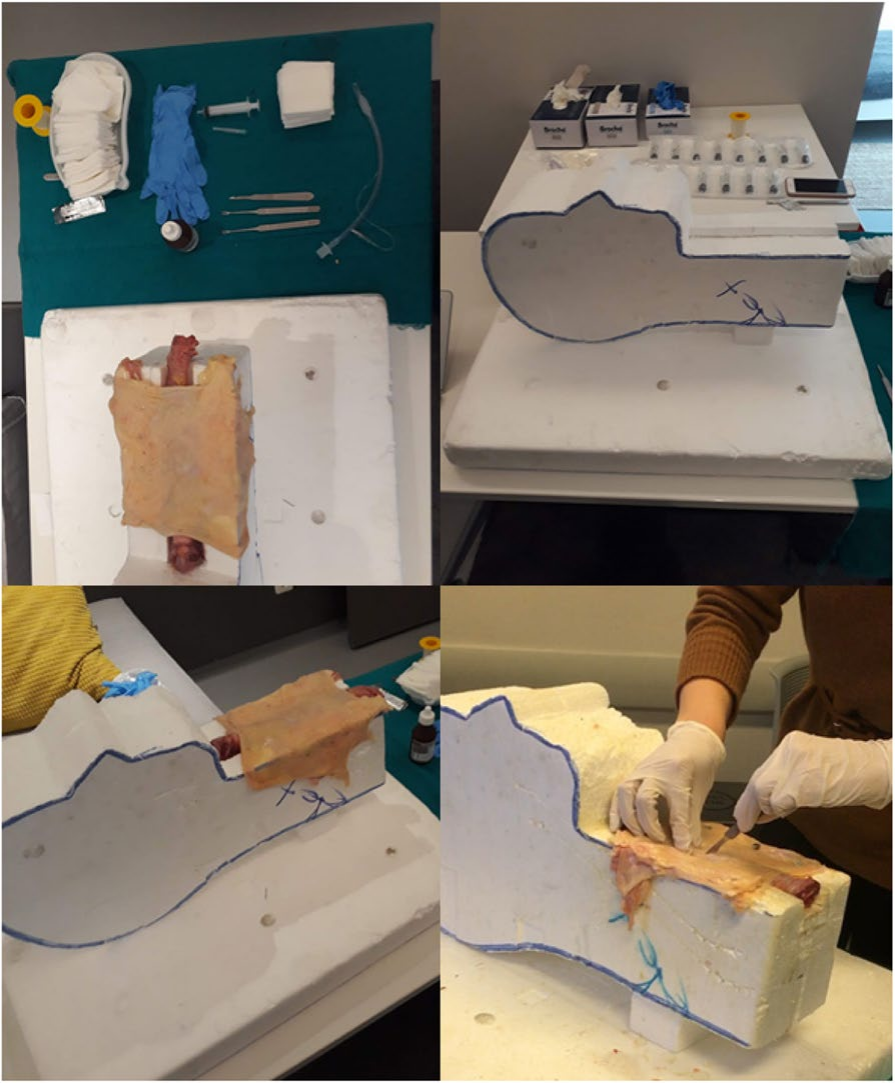
Cricothyroidotomy practice on a realistic mannequin model

We used a simulation model for cricothyroidotomy, which included the use of styrofoam pieces, a sheep trachea, and two layers of chicken skin. Sheep trachea and chicken skin were not extracted from live animals, they were obtained from the slaughterhouse. Cricothyroidotomy was carried out by the trainees with the use of gloves, a scalpel, a scalpel handle, a hook, an endotracheal tube, and a syringe. The sheep trachea was placed in the slot created in the styrofoam, and layers of chicken skin were then fixed over the trachea (Fig. 1). The skin tissue was replaced after every 3–4 procedures.

Each trainee performed the cricothyroidotomy twice, and each attempt was recorded using a dome camera (2 Megapixel, 4.8–120 mm) positioned to record an optimal view of the application site. No instructions were provided to the trainees during the procedure.

A video including the best practices for cricothyroidotomy recorded by two emergency medicine assistant professors and an emergency medicine resident [16] was shown three times to the trainees, following a an educational PowerPoint presentation describing cricothyroidotomy.

The trainees were randomized into two groups after seeing the educational presentation and the best practice video. Simple randomization method was used in the study and allocation ratio was 1:1. One of these groups performed the cricothyroidotomy twice and reviewed their own performance via a video recording for 15 minutes between two attempts. This group is the ‘self video feedback group’ (SVFG). The second group, after the first attempt, reviewed their performance together with an emergency medicine specialist who provided additional feedback about the mistakes made, causes for the mistakes, and the ways to prevent them the next time they perform the procedure. This second group of trainees made up the ‘expert-assisted video feedback group’ (EVFG). The best practice video was not used during the feedback sessions for any of the two groups.

**Fig. 2:**
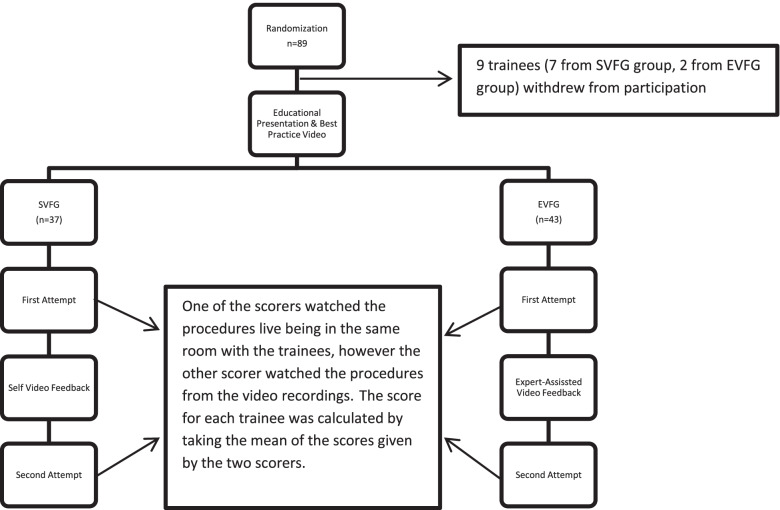
Flow chart summarizing the study process

In order to assess the impact of review and feedback sessions which took place between two attempts on the trainees’ performance, each attempt was scored by two emergency medicine specialists, one who watched the procedure live and the other from the video recording. The scorers used a checklist for the steps of cricothyroidotomy, which was used previously by Ozkaya et al. [13] (Appendix A). These two emergency medicine specialists (scorers) were briefed about the procedure they would evaluate and the checklist they would use. Each step of cricothyroidotomy on the checklist was scored separately. The steps that were performed incorrectly or missed were given the score “1″. Steps which were executed following a pause after the previous step but performed properly and timely were scored as “2″. The score of “3″ was given to those steps which were performed properly, timely, and without any pause or hesitation. The last item on the checklist to be scored was the total duration of the procedure. A threshold of 40 seconds was used, as was done by Wong et al. [17]. The procedures which took up to 40 seconds and more than 40 seconds were scored as “1″ and “2″, respectively. As mentioned above, following the review/feedback sessions (SVFG or EVFG), trainees performed the procedure for a second time, it was recorded and scored by the two scorers. One of the scorers watched the procedures live being in the same room with the trainees, however the other scorer watched the procedures from the video recordings. This second scorer was not able to see the faces of the trainees and could watch the recordings as many times as he wanted. Second scorer, who made the assessment via the video recording was able to make a more blinded/objective assessment due to the fact that he or she could not see the face of the participant. It also allowed us to compare the results and see whether a scorer physically present is necessary. There was a good correlation between the scores given by the two scorers, with a correlation coefficient of 0.83. The score for each trainee was calculated by taking the mean of the scores given by the two scorers. The mean scores of the 1st and the 4th steps on the checklist were also calculated seperately because those are considered as the most critical ones. A summary of the flow of the study steps can be found in Fig. 2.

The study had between 58.8–94.6% power to produce a significant difference with *N* = 80 participants in terms of L Ass.-V Ass._ MEAN, TIME, Step1Step4_Mean with 0.05 type 1 error, and the mean of power is found as 73.9.

### Data analysis

Statistical analysis was performed using the MedCalc Statistical Software version 12.7.7 (MedCalc Software bvba, Ostend, Belgium; http://www.medcalc.org; 2013). Descriptive statistics were presented using mean, standard deviation, median, minimum, maximum scale variables. For comparison of two non-normally distributed independent groups, Mann Whitney U test was used. For comparison of two normally distributed independent groups, Student t-test was used. For comparison of two non-normally distributed dependent groups, the Wilcoxon test was used. For comparison of two normally distributed dependent groups, Paired Samples t-test was used. Statistical significance was accepted when the two-sided *p*-value was lower than 0.05. “Cohen’s d” was used to calculate the effect sizes.

## Results

Data was collected between 10/09/2019–28/11/2019. The study was completed by 80 trainees, since nine withdrew from participation before the procedures were completed. Among these, 37 and 43 were randomized to the SVFG and EVFG groups, respectively. The average age of the trainees was 25. Forty-four (55%) of the trainees were men, and 36 (45%) were women.

The score (mean of the scores given by two scorers) and duration of pre-feedback and post-feedback attempts for both SVFG and EVFG groups are summarized in Table 1. The mean score and duration of the pre-feedback attempt were similar between the two groups. There was a statistically significant improvement of scores between pre-feedback and post-feedback attempts in both SVFG and EVFG groups (*p* < 0.001). However, the post-feedback score was significantly higher in the EVFG group compared with the SVFG group (*p* < 0.05). It is important to note that, apart from being statistically significant, the 3.3-point improvement in how the procedure was performed observed in the SVFG group can be considered as important, given the maximum possible score that can be received from the scoring system used in our study is 29.

**Table 1:** Comparisons according to groups and measurements

	SVFG/EVFG	PreFAss.	PostFAss.	Cohen’s d (Effect size)	***p***
		Mean **+** Std.Dev.	Mean **+** Std.Dev.		
**L Ass.-V Ass._ MEAN**	**SVFG**	19.2 + 3.1	22.5 + 3.6	0.982	**< 0.001**^**1**^
	**EVFG**	19.5 + 3.8	24.3 + 2.9	1.420	**< 0.001**^**2**^
	**Cohen’s d (Effect size)**	0.086	0.550		
	**p**	0.634^3^	**0.027**^**4**^		
**TIME**	**SVFG**	86 + 27	68 + 22	0.730	**< 0.001**^**1**^
	**EVFG**	88 + 37	53 + 14	1.251	**< 0.001**^**1**^
	**Cohen’s d (Effect size)**	0.061	0.813		
	***p***	0.762^3^	**0.001**^**3**^		

*PreFAss* Pre Feedback Assessment, *PostFAss* Post Feedback Assessment, *SVFG* Self Video Feedback Group, *EVFG* Expert Assissted Video Feedback Group, *L Ass* Live Assessment Score, *V Ass* Video Assessment Score

Paired Samples t test^1^,Wilcoxon test^2^,Student t test^3^,Mann-Whitney U test^4^

The time needed to perform the cricothyroidotomy significantly decreased following the feedback session between the two attempts in both groups (*p* < 0.001). The time needed for the procedure was significantly shorter in the EVFG group compared with the SVFG group (*p* = 0.001).

When we compared the mean scores given for the two critical steps (Steps 1 and 4) of the procedure between the groups, the trend was similar with the total score. There was a significant improvement between pre-feedback and post-feedback attempts in both groups (*p* < 0.001), with post-feedback score being significantly higher in the EVFG group compared with the SVFG group (*p* < 0.05) (Table 2).

**Table 2:** Comparisons according to groups (Step1Step4_Mean Differences)

		PreFAss.	PostFAss.	Cohen’s d (Effect size)	***p***
		Mean **+** Std.Dev.	Mean **+** Std.Dev.		
**Step1Step4_Mean**	**SVFG**	1.98 + 0.41	2.35 + 0.55	0.762	**< 0.001**^**1**^
	**EVFG**	1.97 + 0.43	2.59 + 0.41	1.475	**< 0.001**^**1**^
	**Cohen’s d (Effect size)**	0.023	0.494		
	***p***	0.920^2^	**0.047**^**2**^		

Wilcoxon test^1^, Mann-Whitney U test^2^

## Discussion

The traditional model for simulation-based training includes post-simulation feedback in which students’ experiences are discussed and reflected upon to improve future performance [18]. A better understanding of the role of feedback in competency-based education could result in a more efficient learning process for the trainees [19]. In the traditional model, an expert is needed for the post-simulation feedback session. Transitioning the primary responsibility from an expert to the trainee’s self through self-guided learning has received increasing attention, mainly due to the potential of reducing the resources needed [18].

This study was conducted to assess the potential utility of a lower-cost simulation-based method in the education of medical students to perform cricothyroidotomy. The results demonstrated significant improvement in the ability to perform cricothyroidotomy with both self and expert-assisted video feedback. This finding confirms Hawkins’s findings, which demonstrated improvement with video self-assessment (including video recorded ‘benchmark performance’) and expert assessor groups [8].

The results from studies conducted by Nesbitt et al. [20], Backstein et al. [21], and Phillips et al. [10], which demonstrated a significant improvement in the performance of the participants utilizing self video feedback with or without an expert, are also in alignment with our findings. In these three studies, no significant additional benefit was demonstrated from the inclusion of an expert in the feedback process, and a similar improvement was obtained with the use of a best practice expert video and allowing attendees to assess their own performances.

Our results demonstrated significant improvement both in the scores received and the time needed for the cricothyroidotomy with both expert-assisted video feedback and self video feedback methods. This suggests that providing the opportunity to the trainee to review his or her own performance using video feedback is beneficial in improving the learning experience. However, the improvement was significantly greater with the expert-assisted video feedback, which indicates an additional benefit from receiving help from an expert for the the feedback. The results were similar when the two most critical steps of the cricothroidotomy were analyzed separately from the whole procedure.

The results from our study support the idea that even though expert-assisted video feedback is giving better results, self video feedback is able to achieve improvement in cricothyroidotomy performance. Another significant finding is that there was a significant improvement only after a single self video feedback session. In 2003, Wong et al. demonstrated that by self video feedback alone, the 96% of the trainees were able to perform the cricothyroidotomy successfully by the fifth attempt. Although the trainees in Wong et al’s study, who were anesthesiologists, performed the procedure 10 times, which is a considerably higher number when compared with our study which included two attemps, the younger (< 44 years) sub-population in Wong et al’s study had a 90% success rate after only two attemps. This supports the use of two attempts in our study design [17].

An important limitation of this study was that students performed the procedure only twice. In the literature, a higher number of attempts (up to 10) are being mentioned. However, as mentioned above, in the study by Wong et al., 90% of the trainee group who were younger than 44 years achieved success after two trials, which supports our methodology. Also, we didn’t evaluate retention of the capability to perform cricothyroidotomy after a certain period of time following the training. This was mainly due to the limited access to the trainess, some of whom had left after graduating from the medical school. One other limitation is that the study design did not include a control group which did not receive any feedback.

In the light of our findings as well as the previous findings from the literature, it can be stated that self-feedback is a valuable method in the training of students for cricothyroidotomy even though expert-assisted feedback seems to have additional value. This finding is important, especially regarding the circumstances in which expert-assisted feedback cannot be utilized due to resource limitations. Further studies are needed in order to understand the self-feedback method which will lead to maximal improvement in the cricothyroidotomy performance.

## Conclusion

This study demonstrates significant improvement in cricothyroidotomy performance with both self and expert-assisted video feedback methods. This indicates that even though expert-assisted feedback may be delivering better results, self feedback can be a valuable method, especially in resource-limited environments. Additional studies are needed to understand the self-feedback method which will provide most optimal results.

## Supplementary Information

**Additional file 1.**

## Data Availability

All data generated or analysed during this study are included in this published article (and its supplementary information files).
