# Supplementary material for: Comparison of self versus expert-assisted feedback for cricothyroidotomy training: a randomized trial
Source: BMC Med Educ. 2022 Jun 14;22:455. doi: 10.1186/s12909-022-03519-z (PMC9199165; doi:10.1186/s12909-022-03519-z)
Supplement: Supplementary file 1 — Additional file 1. [file 12909_2022_3519_MOESM1_ESM.docx]

**Appendix A**

The cricothyroidotomy skill checklist used in the study

Steps required for the procedure:

1. Localizing of the anterior part of the cricothyroid membrane between the thyroid and cricoid cartilage.

2. Stabilizing the trachea with the non-dominant hand.

3. Applying a midline vertical skin incision of 2 to 3 cm from the caudal end of the thyroid cartilage to the cephalic end of the cricoid cartilage.

4. Making a transverse incision of 1 to 2 cm from the crythyroid membrane.

5. Inserting the scalpel handle into the incision and rotating it 90°.

6. Inserting a tracheal hook into the incision and hooking the caudal end of the opening.

7. Allowing for the passage of a cuffed endotracheal tube (usually No. 5 or No. 6), directing the tube distally.

8. Inflating the cuff.

9. Securing the airway.

10. Completing the procedure in 40 seconds.
